# Serotype Distribution and Antimicrobial Resistance of *Streptococcus pneumoniae* Invasive Isolates Collected at the Italian Hospital of Desio, Lombardy, from 2008 to 2016

**DOI:** 10.3389/fpubh.2017.00169

**Published:** 2017-07-14

**Authors:** Jari Intra, Silvia Besana, Cinzia Savarino, Paolo Brambilla

**Affiliations:** ^1^Department of Laboratory Medicine, University of Milano-Bicocca, Desio Hospital, Desio, Italy

**Keywords:** pneumonia, meningitis, sepsis, pneumococcal vaccine, antibiotic

## Introduction

The encapsulated Gram-positive bacterium *Streptococcus pneumoniae* is a major pathogen that causes pneumonia, meningitis, and acute otitis media in children, elderly people, and immunocompromised population, with high morbidity and mortality (1). In many developed countries, pneumococcal infections interest an increasing number of patients affected by chronic disease, such as diabetes, asthma, chronic obstructive pulmonary disease, cardiovascular disease, chronic renal failure, and sickle-cell disease (1). Approximately 30% of adult pneumonia cases are caused by *S. pneumoniae* with a mortality rate between 11 and 40%, and 70–100 million of children aged under 5 years die for pneumococcal disease annually (2). For this reason, over the past 30 years, many efforts have been made to reduce the rate of pneumococcal infection by vaccination. Three multivalent pneumococcal conjugate vaccines have been developed to reduce the disease caused by specific pneumococcal serotypes in numerous countries (3–5). The first 7-valent pneumococcal polysaccharide conjugate vaccine (PCV7) developed in 2000 contains capsular polysaccharides from seven serotypes (4, 6B, 9V, 14, 18C, 19F, and 23F) and it significantly reduced the rate of infections in children under 2 years (1). The second and third conjugate vaccines 10-valent (PCV10) and 13-valent (PCV13) were developed in 2009 and in 2010, respectively. They contain the seven serotypes of PCV7 (4, 6B, 9V, 14, 18C, 19F, and 23F) and three serotypes (1, 5, and 7F) for PCV10, five serotypes (1, 3, 5, 7F, and 19A) and 6A for PCV13. Protective benefit against pneumococcal infection was increased (1, 6). The polysaccharide non-conjugate vaccine PPV23, developed in 1983, contains purified capsular polysaccharides from 23 serotypes (1, 2, 3, 4, 5, 6B, 7F, 8, 9N, 9V, 10A, 11A, 12F, 14, 15B, 17F, 18C, 19A, 19F, 20, 22F, 23F, and 33F), and it is recommended for adults aged 65 years and older. However, it is not used for children younger than 2 years old (1). In Italy, PCV7 was used until 2010, and then it was substituted by PCV13. PCV vaccination is offered to all infants younger than 3 years, while no specific suggestion has been given to adults.

Prospective, multicentre, and observational studies are desirable to help the health Authorities in the development of efficient immunization strategies. The World Health Organization recommends countries to carry out appropriate surveillance of pneumococcal infections in order to estimate the vaccine coverage rate and to observe the effect of vaccination (7, 8). In Italy, few studies have been conducted to estimate the status of pneumococcal disease among children and adults (9–11). Recently, a prospective study has been conducted in children younger than 5 years in North-West Lombardy, including the city of Milan (12).

The increase of the presence of multi-drug resistant (MDR) *S. pneumoniae* has been observed in many countries and it is becoming a main problem worldwide (13, 14). The national immunization programs and the clinical importance of vaccines can substantially decrease the infection rate of *S. pneumoniae* and the emergence of antibiotic-resistant pneumococci (13, 14).

The main goal of this study was to describe the serotype distribution, antimicrobial susceptibility, and resistance of invasive *S. pneumoniae* strains isolated from pediatric and older patients admitted to the hospital of Desio (MB), Italy. These data allow monitoring the status of vaccination, guiding the use of different vaccines both in children and the elderly, and suggesting the development of a new generation of conjugate vaccines.

## Methods

### Clinical Isolates and Culture Conditions

This study was conducted at the Desio Hospital, situated in the Italian region of Lombardy with around 9,000,000 resident people. Starting from the large computerized database of the hospital, 60 non-duplicated patients with a confirmed diagnosis of invasive pneumococcal disease in the period June 2008–March 2016 were included in this work (Data Sheet S1 in Supplementary Material). In accordance with the Centers for Disease Control and Prevention (Atlanta, GA, USA), a case of invasive pneumococcal disease was defined as the isolation of a *S. pneumoniae* strain from clinical specimens of normally sterile body sites, such as blood or cerebral spinal fluid (CSF). Samples were inoculated on 5% sheep blood agar plates (bioMerieux, La Balme-les-Grottes, France), and incubated at 37°C in the presence of 5% CO_2_ for 18–24 h prior the identification.

### Identification of Isolates

*Streptococcus pneumoniae* isolates were analyzed and identified using standardized laboratory procedures, including colony morphology on blood agar, the presence of alpha-hemolysis, the optochin sensitivity test (Oxoid, Basingstoke, UK), and the catalase test, as described by UK Standards for Microbiology Investigations. Conventional microbiological methods were performed in combination with the Matrix Assisted Laser Desorption Ionization Time-of-Flight VITEK^®^ MS and the VITEK^®^ 2 microbial analysis systems, in accordance with the manufacturer’s instructions (bioMerieux, La Balme-les-Grottes, France).

### Susceptibility Testing

The antimicrobial susceptibility of each strain was performed using the VITEK^®^ 2 *S. pneumoniae* susceptibility card (AST-P576, bioMerieux), and the interpretation of the results complied with the accordance to the European Committee on Antimicrobial Susceptibility Testing breakpoints tables (15).

### Serotyping of Isolates

All isolates were subcultured and then they were sent in two Amies medium swab to the Pneumococcal Reference Laboratory in Lombardy (Milan, Italy) for analysis. At reference laboratory, each isolate was initially confirmed as *S. pneumoniae*, and then serotyped. Serogrouping was carried out using a commercial kit based on latex agglutination (Pneumotest-Latex kit, Statens Serum Institut, Copenhagen, Denmark). Briefly, pneumococcal serogroups were identified using latex particles coated with rabbit antibodies raised against capsular polysaccharide of pneumococci after agglutination in 14 pool sera and 21 group sera.

After group identification, *S. pneumoniae* serotypes was performed using Omni serum (Statens Serum Institut) that includes antibodies to all known pneumococci. Briefly, a suspension of pure cultures of capsulated pneumococci was mixed with one drop of type antiserum and one drop of methylene blue on a glass slide. After an incubation at room temperature for 10 min, the samples were observed at microscope, and if the capsule was visible, the reaction was considered positive (Quellung reaction).

## Results

Sixty *S. pneumoniae* strains were collected from patients with diagnosis of invasive pneumococcal infection at Desio Hospital, Lombardy (Italy), from January 2008 to March 2016. Our patient’s age ranged from 5 to 96 years, with an average of 59 ± 28 years. The male-to-female ratio was 1.31:1 (males = 56.7%; females = 43.3%). The most prevalent source was blood (*n* = 40, 67%), and then CSF (*n* = 9; 15%). Eleven patients (18%) showed pneumococcal infection in both blood and CSF samples. Sixty-eight percent of pneumococcal infections collected in this study are sepsis, while 32% are meningitis. Our data agreed to the incidence in Italy (16). Among the 60 strains isolated, nine (15%) were isolated from patients aged between 5 and 12 years old (*n* = 3 preschooler; *n* = 6 schooler), none in patients aged between 13 and 27 years, 16 (26.7%) were isolated from patients between 28 and 64 years, 35 (58.3%) were isolated from patients ≥65 years. It is important to note that no *S. pneumoniae* strains were isolated from children younger than 5 years.

**Figure 1:**
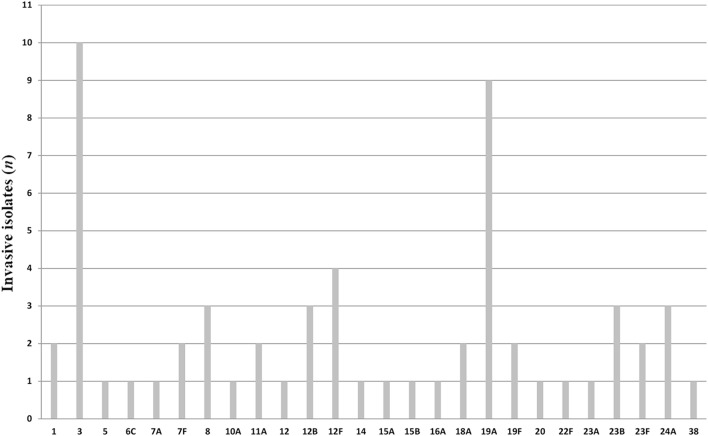
Serotype distribution of *Streptococcus pneumoniae* isolates.

All pneumococci isolated were identifiable by capsular serotyping. Figure 1 showed the serotype distribution of *S. pneumoniae* strains. The most common isolated serotypes were 3 (*n* = 10, 16.7%), 19A (*n* = 9, 15%), and 12F (*n* = 4, 6.7%), accounting for 38.3% among the isolates. In patients aged 5–12, serotypes 1, 12F, 19A, 23B, and 38 accounted 100% of serotyped isolates (Table 1). All the serotypes caused sepsis, and only in two cases, serotypes 19A and 23B caused both sepsis and meningitis. Sixteen serotypes were isolated in patients aged 13–64 years: eight caused sepsis, five caused meningitis, and three caused both sepsis and meningitis. The most observed serotypes were 19A and 19F, which accounted 25% of isolates in this group (Table 1). In individuals older than 65 years, 17 serotypes were isolated and serotyped. The most prevalent strains were 3 (*n* = 9) and 19A (*n* = 5), which represented the 40% (Table 1).

**Table 1:** Serotype distribution among clinical specimens and age groups.

Pneumococcal serotypes	Years		
	5–12	13–64	>65
1	2		
3		1	9
5		1	
6C			1
7A			1
7F		1	1
8			3
10A		1	
11A		1	1
12		1	
12B			3
12F	2		2
14		1	
15A		1	
15B			1
16A			1
18A		1	1
19A	2	2	5
19F		2	
20			1
22F			1
23A			1
23B	2	1	
23F		1	1
24A		1	2
38	1		

It is worth noting that there were some association between *S. pneumoniae* strains and the type of invasive infection. We observed that the 90% of isolates belonging to serotype 3 and 100% of isolates belonging to serotype 8 caused sepsis in patients older than 65 years, while the only serotype 3 isolated in a patient aged 40 years caused meningitis. One hundred percent of isolates belonging to serotype 1 caused sepsis only in children. Moreover, all the clinical cases of pneumococcal infections due to serotype 24A caused meningitis in patients older than 12 years.

Evaluating the antimicrobial susceptibility, we noted that all strains were susceptible to cefotaxime, ceftriaxone, imipenem, levofloxacin, moxifloxacin, ofloxacin, linezolid, vancomycin, chloramphenicol, rifampicin, and trimethoprim/sulfamethoxazole. Pneumococcal serotypes identified in patients younger than 12 years were susceptible to all the tested antibiotics. On the contrary, five serotypes (14, 15A, 19A, 19F, and 24A) identified in patients aged 13–64 years showed antimicrobial resistance to erythromycin [minimum inhibitory concentration (MIC) ≥ 1 µg/ml] and tetracycline (MIC ≥ 16 µg/ml) and two serotypes (15A and 24A) were also resistant to penicillin (MIC ≥ 2 µg/ml). In individuals older than 65 years, seven serotypes (3, 6C, 15B, 19A, 20, 23F, and 24A) were resistant to erythromycin (MIC ≥ 1 µg/ml) and tetracycline (MIC ≥ 16 µg/ml) and the strain 24A was also resistant to penicillin (MIC ≥ 2 µg/ml).

Importantly, we found that potential immunization coverage rates of PCV13, estimated on typified pneumococcal serotypes across all the period of this study, were 44.4% for children between 5 and 12 years, 56% for patients between aged 13 and 64 years, and 46% for patients older than 65 years. On the other hand, potential coverage rates were higher using the non-conjugate 23-valent pneumococcal polysaccharide vaccine: 67, 68.7, and 71.4%, respectively.

## Discussion

In 2014, in Italy, the overall incidence of confirmed invasive pneumococcal disease was 1.57/100,000 (16). Incidence was 3.34/100,000 in infants (0 years), 1.44/100,000 in preschooler (1–4 years), 0.28–0.84/100,000 in schooler (5–14 years), 0.15–1.03/100,000 in patients aged 15–64 years, and 3.99/100,000 in patients aged >64 years (16). In 2014, in the regions of northern Italy (including Lombardy) annual incidence grew to 5.86/100,000 in infants and 8.31/100,000 in individuals older than 64 years, while it was similar to national data in patients aged 5–64 years (16). Our study provides data for a continuous surveillance of *S. pneumoniae* strains causing invasive pneumococcal diseases, and antibiotic resistance patterns in order to evaluate the constant effect of vaccines and their possible useful development (1).

More than 90 serotypes of *S. pneumoniae* exist, but only a subset causes invasive disease. *S. pneumoniae* isolates collected worldwide from 2004 to 2009 showed different geographical distribution (17). In this study, we identified 26 serotypes causing infections. The serotypes 3 and 19A are the most found, in agreement with the findings from recent Italian pooled data obtained by the National Institute of Health (2013) (16). In particular, the serotype 19A, which accounted 15% of our serotyped isolates, was indicated as an emerging strain. In fact, although it is included in PCV13 vaccine and not in PCV7, its increasing frequency has been recently demonstrated in Italy and worldwide (12, 18–21).

The distribution and antimicrobial resistances of *S. pneumoniae* serotypes were reported in different studies (17, 21–26). Recently, in Italy, it has been presented a work on a large population of two regions (Friuli-Venezia Giulia and Tuscany), evaluating the serotype distribution of *S. pneumoniae* isolates in children and adults. This study demonstrated a low correspondence between pneumococcal serotypes found in children and serotypes found in adults (11). In agreement with these data, in our study, we observed that 100% of infections caused by serotypes 3, 7F, 8, 19F, 23F, 24A and 78% of infections caused by serotype 19A are associated with invasive pneumococcal infections in adults, in particular in patients older than 65 years. Conversely, 100% of invasive infections caused by serotype 1 were detected in children. This phenomenon is unexpected, since it has been demonstrated that some serotypes are frequently invasive, while others are commonly found in carriers (11).

In this study, we observed that invasive pneumococcal infections in children younger than 5 years of age were not detected and only five *S. pneumoniae* serotypes were isolated from patients aged 5–12 years, demonstrating that current regional vaccination programs effectively reduce invasive infections. In fact, among the nine cases identified, only a 5-year-old patient contracted an invasive pneumococcal infection by serotype 1, which is included in PCV13 vaccine. Moreover, three patients, who contracted infection by serotypes 1 and 19 A, were born before the introduction of PCV13 vaccine and they received PCV7. These data confirmed the substantial benefits and improvements after the introduction of PCV13 (1). Considering invasive pneumococcal infection in adults, we observed a progressive advance of incidence with increasing age. PCV vaccine is included in the Italian national program of vaccination in infants, while no definite suggestion has been specified to adults. Each Italian region follows different strategies. In this case, in Lombardy, only patients with particular pathologies, such as defects of the immune system, chronic illnesses, and diabetes, are recommended to receive PCV vaccination. As recently demonstrated, PCV13 vaccination of children seems to produce a small impact in the reduction of pneumococcal infections in adults (11, 27). In fact, in our study, the most common serotypes found, e.g., 3 and 19A, although they are included in PCV13, caused diseases in adults. Moreover, more recently, it has been shown that revaccination of adults older than 50 years with PCV13 after a first vaccination administered 5 years earlier stimulates a memory response, maintaining higher antibody titers (28). According to our data, we suggest that a specific vaccination program might be offered to adults in order to decrease invasive pneumococcal disease. In fact, PPV23 seems to be a good solution since about 70% of adults might be covered. However, further works and active surveillance studies are necessary in order to reveal the potential beneficial effects of PPV23 in immunization programs of children older than 5 years and adults to decrease disease burden in the population, as also suggested by World Health Organization (29).

Over the past years, inadequate prescription and consumption of antibiotics caused an increasing incidence of antimicrobial-resistant pneumococci, leading to a global problem (13, 14). In this study, not very low representation of antibiotic resistance was observed. Resistance to inhibitors of protein synthesis was observed in the highest percentage (26.7%), while the lowest percentage was observed for cell wall synthesis inhibitors, in particular penicillin (5%). Moreover, the serotype 24A isolated from three adults is multiresistant (MDR) with combined non-susceptibility to penicillin and erythromycin, most probably carried by transposons of the Tn916 family, as recently described (30). Similar results to our data were recently observed in a study performed in Italy (31). Complex relationships between prevalence of resistance and incautious antibiotic use suggest that serotypes distribution also depends on the timing of antimicrobial administration. Vaccination programs not only lead to a very high reduction of invasive pneumococcal disease but also reduce an incorrect use of antibiotics.

Finally, a limitation of this study is the restricted numbers of strains isolated in one hospital. Therefore, multicentre studies, including isolates from more hospitals, should be carried out.

*Streptococcus pneumoniae* is an important cause of invasive diseases, in particular in children and elderly. The results obtained in this study provided important data on serotypes distribution and antibiotic resistances. Continued surveillance of pneumococcal epidemiology is strongly suggested, making available information on the emergence of multi-drug-resistant strains. In conclusion, this work could be an aid to monitor the dynamic changing of serotypes and antimicrobial resistances, suggesting a clinical guidance for the development of appropriate antimicrobial therapies and for the inclusion of emergent serotypes in a new generation of vaccines.

## Ethics Statement

This article did not contain any studies with human participants and/or animals.

## Author Contributions

JI and PB designed the study. JI, SB, and CS collected the data, performed the analysis, and wrote the manuscript. JI, SB, CS, and PB approved the final manuscript.

## Conflict of Interest Statement

The authors declare that the research was conducted in the absence of any commercial or financial relationships that could be construed as a potential conflict of interest.

## Supplementary Material

Data Sheet S1 Data set used in this study contains information about year of isolation of *Streptococcus pneumoniae* strain, patient age at the time of detection, patient gender, serotype of pneumococcal strains isolated, antibiotic resistance [name of antimicrobial agent to which a *S. pneumoniae* strain showed resistance and minimum inhibitory concentration (MIC) value], and tissue site of *S. pneumoniae* infection.

## References

[1] Daniels CC Rogers PD Shelton CM. A review of pneumococcal vaccines: current polysaccharide vaccine recommendations and future protein antigens. J Pediatr Pharmacol Ther (2016) 21:27–35. 10.5863/1551-6776-21.1.27 26997927 PMC4778694

[2] Nuorti JP Whitney CG Centers for Disease Control and Prevention (CDC) Prevention of pneumococcal disease among infants and children – use of 13-valent pneumococcal conjugate vaccine and 23-valent pneumococcal polysaccharide vaccine – recommendations of the Advisory Committee on Immunization Practices (ACIP). MMWR Recomm Rep (2010) 59:1–18. 21150868

[3] Chiba N Morozumi M Shouji M Wajima T Iwata S Ubukata K Changes in capsule and drug resistance of pneumococci after introduction of PCV7, Japan, 2010–2013. Emerg Infect Dis (2014) 20:1132–9. 10.3201/eid2007.131485 24960150 PMC4073837

[4] Mendes RE Costello AJ Jacobs MR Biek D Critchley IA Jones RN. Serotype distribution and antimicrobial susceptibility of USA *Streptococcus pneumoniae* isolates collected prior to and post introduction of 13-valent pneumococcal conjugate vaccine. Diagn Microbiol Infect Dis (2014) 80:19–25. 10.1016/j.diagmicrobio.2014.05.020 24974272

[5] Navarro Torné A Dias JG Quinten C Hruba F Busana MC Lopalco PL European enhanced surveillance of invasive pneumococcal disease in 2010: data from 26 European countries in the post-heptavalent conjugate vaccine era. Vaccine (2014) 32:3644–50. 10.1016/j.vaccine.2014.04.066 24795228

[6] Bryant KA Block SL Baker SA Gruber WC Scott DA PCV13 Infant Study Group Safety and immunogenicity of a 13-valent pneumococcal conjugate vaccine. Pediatrics (2010) 125:866–75. 10.1542/peds.2009-1405 20435707

[7] Deloria Knoll M Park DE Johnson TS Chandir S Nonyane BA Conklin L Systematic review of the effect of pneumococcal conjugate vaccine dosing schedules on immunogenicity. Pediatr Infect Dis J (2014) 33:S119–29. 10.1097/INF.0000000000000079 24336054 PMC3940378

[8] Said MA Johnson HL Nonyane BA Deloria-Knoll M O’Brien KL AGEDD Adult Pneumococcal Burden Study Team, et al. Estimating the burden of pneumococcal pneumonia among adults: a systematic review and meta-analysis of diagnostic techniques. PLoS One (2013) 8:e60273. 10.1371/journal.pone.0060273 23565216 PMC3615022

[9] Tarallo L Tancredi F Schito G Marchese A Bella A Italian Pneumonet Group (Società Italiana Pediatria and Associazione Italiana Studio Antimicrobici e Resistenze). Active surveillance of *Streptococcus pneumoniae* bacteremia in Italian children. Vaccine (2006) 24:6938–43. 10.1016/j.vaccine.2006.05.012 16901591

[10] Tardivo S Poli A Zerman T D’Elia R Chiamenti G Torri E Invasive pneumococcal infections in infants up to three years of age: results of a longitudinal surveillance in North-East Italy. Ann Ig (2009) 21:619–28. 20169833

[11] Azzari C Cortimiglia M Nieddu F Moriondo M Indolfi G Mattei R Pneumococcal serotype distribution in adults with invasive disease and in carrier children in Italy: should we expect herd protection of adults through infants’ vaccination? Hum Vaccin Immunother (2016) 12:344–50. 10.1080/21645515.2015.1102811 26647277 PMC5049737

[12] Riva E Salvini F Garlaschi ML Radaelli G Giovannini M The status of invasive pneumococcal disease among children younger than 5 years of age in north-west Lombardy, Italy. BMC Infect Dis (2012) 3:106 10.1186/1471-2334-12-106 PMC3406943 22554011

[13] Liñares J Ardanuy C Pallares R Fenoll A Changes in antimicrobial resistance, serotypes and genotypes in *Streptococcus pneumoniae* over a 30-year period. Clin Microbiol Infect (2010) 16:402–10. 10.1111/j.1469-0691.2010.03182.x 20132251

[14] Karcic E Aljicevic M Bektas S Karcic B. Antimicrobial susceptibility/resistance of *Streptococcus pneumoniae*. Mater Sociomed (2015) 27:180–4. 10.5455/msm.2015.27.180-184 26236165 PMC4499292

[15] The European Committee on Antimicrobial Susceptibility Testing. Breakpoint Tables for Interpretation of MICs and Zone Diameters (2016). Available from: http://www.eucast.org

[16] ISS. Dati di sorveglianza delle malattie batteriche invasive aggiornati al 4 aprile 2016 (2016). Available from: http://www.iss.it/binary/mabi/cont/Report_MBI_20160404.pdf

[17] Hackel M Lascols C Bouchillon S Hilton B Morgenstern D Purdy J. Serotype prevalence and antibiotic resistance in *Streptococcus pneumoniae* clinical isolates among global populations. Vaccine (2013) 31:4881–7. 10.1016/j.vaccine.2013.07.054 23928466

[18] Hsieh YC Huang YC Lin HC Ho YH Chang KY Huang LM Characterization of invasive isolates of *Streptococcus pneumoniae* among Taiwanese children. Clin Microbiol Infect (2009) 15:991–6. 10.1111/j.1469-0691.2009.02743.x 19392891

[19] Reinert R Jacobs MR Kaplan SL. Pneumococcal disease caused by serotype 19A: review of the literature and implications for future vaccine development. Vaccine (2010) 28:4249–59. 10.1016/j.vaccine.2010.04.020 20416266

[20] Skoczyńska A Sadowy E Bojarska K Strzelecki J Kuch A Gołębiewska A Participants of laboratory-based surveillance of community acquired invasive bacterial infections (BINet). The current status of invasive pneumococcal disease in Poland. Vaccine (2011) 29:2199–205. 10.1016/j.vaccine.2010.09.100 20943207

[21] Pan F Han L Huang W Tang J Xiao S Wang C Serotype distribution, antimicrobial susceptibility, and molecular epidemiology of *Streptococcus pneumoniae* isolated from children in Shanghai, China. PLoS One (2015) 10:e0142892. 10.1371/journal.pone.0142892 26571373 PMC4646667

[22] Tai SS. *Streptococcus pneumoniae* serotype distribution and pneumococcal conjugate vaccine serotype coverage among pediatric patients in East and Southeast Asia, 2000–2014: a pooled data analysis. Vaccine (2016) 4:E4. 10.3390/vaccines4010004 26907356 PMC4810056

[23] Ochoa-Gondar O Figuerola-Massana E Vila-Corcoles A Aguirre CA de Diego C Satue E Epidemiology of *Streptococcus pneumoniae* causing acute otitis media among children in Southern Catalonia throughout 2007–2013: incidence, serotype distribution and vaccine’s effectiveness. Int J Pediatr Otorhinolaryngol (2015) 79:2104–8. 10.1016/j.ijporl.2015.09.022 26453272

[24] Ramdani-Bouguessa N Ziane H Bekhoucha S Guechi Z Azzam A Touati D Evolution of antimicrobial resistance and serotype distribution of *Streptococcus pneumoniae* isolated from children with invasive and noninvasive pneumococcal diseases in Algeria from 2005 to 2012. New Microbes New Infect (2015) 6:42–8. 10.1016/j.nmni.2015.02.008 26106481 PMC4475694

[25] Ceyhan M Dagan R Sayiner A Chernyshova L Dinleyici EÇ Hryniewicz W Surveillance of pneumococcal diseases in Central and Eastern Europe. Hum Vaccin Immunother (2016) 20:1–11. 10.1080/21645515.2016.1159363 PMC4994721 27096714

[26] Kim SH Song JH Chung DR Thamlikitkul V Yang Y Wang H Changing trends in antimicrobial resistance and serotypes of *Streptococcus pneumoniae* isolates in Asian countries: an Asian Network for Surveillance of Resistant Pathogens (ANSORP) study. Antimicrob Agents Chemother (2012) 56:1418–26. 10.1128/AAC.05658-11 22232285 PMC3294909

[27] Grau I Ardanuy C Cubero M Benitez MA Liñares J Pallares R. Declining mortality from adult pneumococcal infections linked to children’s vaccination. J Infect (2016) 72:439–49. 10.1016/j.jinf.2016.01.011 26868606

[28] Frenck RW Jr Fiquet A Gurtman A van Cleeff M Davis M Rubino J Immunogenicity and safety of a second administration of 13-valent pneumococcal conjugate vaccine 5 years after initial vaccination in adults 50 years and older. Vaccine (2016) 34(30):3454–62. 10.1016/j.vaccine.2016.04.093 27155493

[29] World Health Organization. Pneumococcal vaccines. WHO position paper – 2012 – recommendations. Vaccine (2012) 30:4717–8. 10.1016/j.vaccine.2012.04.093 22621828

[30] Calatayud L Ardanuy C Tubau F Rolo D Grau I Pallarés R Serotype and genotype replacement among macrolide-resistant invasive pneumococci in adults: mechanisms of resistance and association with different transposons. J Clin Microbiol (2010) 48:1310–6. 10.1128/JCM.01868-09 20147647 PMC2849543

[31] Camilli R Daprai L Cavrini F Lombardo D D’Ambrosio F Del Grosso M Pneumococcal carriage in young children one year after introduction of the 13-valent conjugate vaccine in Italy. PLoS One (2013) 8:e76309. 10.1371/journal.pone.0076309 24124543 PMC3790677

